# Acute Psoriatic Exacerbation Secondary to Infected Pacemaker With Staphylococcus aureus and Candida lusitaniae?

**DOI:** 10.7759/cureus.25078

**Published:** 2022-05-17

**Authors:** Ashish Lalani, Caleb Conrad, Shahman Shahab

**Affiliations:** 1 Internal Medicine, Unity Health, Little Rock, USA; 2 Internal Medicine, Unity Health, Searcy, USA; 3 Osteopathic Medicine, Arkansas College of Osteopathic Medicine, Fort Smith, USA

**Keywords:** pacemaker, vancomycin, infection, red man syndrome, dactylitis

## Abstract

Psoriasis is a lifelong chronic hyperproliferative inflammatory immune-mediated disorder. There is a strong association of psoriasis exacerbation with infection of Staphylococcus and Streptococcus species. In the case of our patient, a psoriatic flare manifesting as dactylitis occurred secondary to methicillin sensitive *Staphylococcus aureus* bacteremia colonization of his pacemaker. If a patient is started on an antibiotic regimen, such as vancomycin, and has symptoms of rash, and swelling in the fingers, it is imperative to ask for a proper rheumatologic history, as vancomycin infusion reaction (previously known as Red Man Syndrome) may not be the cause of the flare-up, such as in the case of our patient. Inversely, patients with psoriasis are more likely to be colonized by *S. aureus* on the skin and in the nasal cavity, and this can lead to bacteremia and infection of hardware, such as an automatic implantable cardioverter-defibrillator (AICD).

## Introduction

Psoriasis is a chronic hyperproliferative inflammatory disease with a prevalence of 2%-3% in the general population [1-3]. It is a lifelong immune-mediated disorder characterized by periods of remission, relapse, and exacerbation. Epidemiological studies suggest multiple factors that can lead to exacerbation of psoriasis including prescription drugs, cutaneous trauma, alcohol, cigarette smoking, stress, and infections [4-9]. There is a strong association of psoriasis exacerbation with infection of streptococcal or staphylococcal species [10]. The influence of antibiotics on the course of psoriasis is controversial, although tetracyclines have been implicated in the exacerbation of psoriasis [11]. Vancomycin infusion reaction (previously known as Red Man Syndrome) can also lead to symptoms similar to a psoriatic flare, such as flushing, erythema, and pruritus [12]. Vancomycin infusion reaction is a rate-related infusion reaction, and if the rate is slowed down, the symptoms generally resolve [12]. 

Psoriatic arthritis is an immune-mediated chronic systemic arthritis with a prevalence of 22%-27% among psoriasis patients [13-14]. Dactylitis, also called sausage fingers, is a hallmark manifestation of psoriatic arthritis characterized by swelling of an entire digit, most commonly the toes. The pathophysiology of dactylitis has been attributed to synovitis, tenosynovitis, enthesitis, and soft tissue edema [15]. The prevalence of dactylitis is between 16% and 49% in patients with psoriatic arthritis [16-17]. 

In addition to psoriatic arthritis, several other conditions can lead to the development of dactylitis. Dactylitis is observed in patients with disorders such as arthritis, tuberculosis, sickle cell disease, sarcoidosis, syphilis, as well as in reactive arthritis [18-19]. Bacterial infections such as those caused by Salmonella, Shigella, Staphylococcus, Yersinia, and Campylobacter can precipitate reactive arthritis leading to dactylitis [8]. Sometimes, these bacterial infections can seed in implanted devices, such as in the case of our patient.

## Case presentation

A 51-year-old male with a past medical history of paroxysmal atrial fibrillation (A. Fib) and chronic congestive heart failure with an implantable cardioverter-defibrillator (ICD) presented to the emergency department for possible ICD incision site infection. He noticed redness, swelling, and pain around the incision site with an associated blister draining straw color fluid two days before presentation to the emergency department. The erythema worsened since onset and increased in diameter, roughly 20 cm by 20cm when seen in the emergency department. He also noticed increased drainage from the associated blister, which was roughly 0.5 cm by 0.5 cm, since symptom onset. He treated the infection with over-the-counter triple-antibiotic (bacitracin zinc - neomycin sulfate - polymyxin B sulfate) ointment with no alleviation of symptoms. He denied any itching, purulent discharge, fever, nausea, or vomiting. He also denied chest pain, shortness of breath, palpitations, headache, or dizziness.

His reported past medical history is significant for chronic systolic congestive heart failure (heart failure with reduced ejection fraction) with a last known ejection fraction of 19%, ICD implant in 2011, with generator change in 2017, and repositioning of right ventricular lead within six months of presentation, paroxysmal A. Fib, chronic obstructive pulmonary disease (COPD), and essential hypertension. He also gave a history of methamphetamine use, marijuana use, and has smoked one pack of cigarettes per day for 14 years.

In the emergency department, blood cultures and a wound culture were drawn, and he was started on vancomycin 15 mg/kg empirically. A cardiology consult was also placed for emergent evaluation. He was admitted for further evaluation and care with the intent to transfer to an institution with a higher level of care for anticipated laser lead extraction due to the increased probability of scarring around ICD leads.

**Figure 1:**
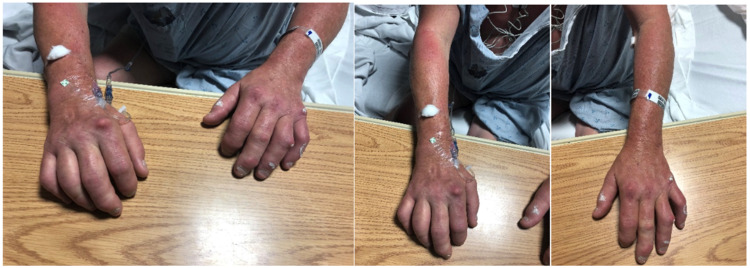
Images of bilateral digit swelling with upper extremity rash, which resolved after removal of pacemaker.

A complete blood count the next morning showed a white blood cell count of 13,100 per microliter decreased from 17,500 per microliter on admission, indicating effective antibiotic therapy. However, he developed a new-onset pruritic, erythematous rash on his upper extremities bilaterally, chest, and neck as well as new-onset red, hot, itch suggestive of the vancomycin infusion reaction and swollen fingers bilaterally, worse on the right as seen in Figure 1 below.

The patient had been on vancomycin for two days when the symptoms of rash, and swelling in the fingers started. Vancomycin was discontinued that same day due to concern for vancomycin infusion reaction; daptomycin 500 mg given over 30 min in 50 mL normal saline IV and piperacillin-tazobactam 3.375 g every six hours IV were started. Upon further questioning, the patient revealed a past medical history of psoriasis that he failed to disclose during the initial encounter. Vancomycin infusion reaction was ruled out, as the patient had a persistent rash and swelling of fingers even after discontinuation of vancomycin, and the patient was started on ibuprofen 800 mg every six hours as needed for dactylitis. He was transferred to a facility with a higher level of care later that day. After transfer, vancomycin was restarted and a CT scan of the right upper extremity two days after the development of dactylitis showed extensive erosive changes throughout the wrist and hand with associated abnormal crystal deposition within multiple flexors and extensor tendons of the hand. He continued ibuprofen 800 mg every six hours for seven days until his dactylitis resolved.

Initial wound cultures from the ICD incision site grew methicillin-susceptible Staphylococcus aureus (MSSA) and blood cultures were positive for one of two cultures drawn for coagulase-negative staphylococcus.

The ICD was successfully removed with subsequent laser lead extraction of right atrial and right ventricular leads, debridement of ICD pocket, and Impella (axial flow pump) placement. The Impella was removed one day after placement with the evacuation of a right chest wall hematoma with thrombectomy of the right subclavian artery. Cultures from the extracted ICD grew MSSA and *Candida lusitaniae*. He was treated with cefazolin 2 g every eight hours IV and micafungin 150 mg once daily IV for 14 days. At discharge, he received a single dose of dalbavancin 1 g IV and was started on cefalexin 500 mg every six hours (to cover for MSSA) taken orally for outpatient antibacterial therapy, and was transitioned to fluconazole 400 mg once daily for outpatient antifungal therapy. He received a second dose of dalbavancin 500 mg IV at a one-week follow-up and was instructed to continue antifungal therapy for an additional two weeks, for a total course of four weeks post-ICD removal.

## Discussion

Infection of a cardiac implantable electronic device (CIED) is the most serious complication of pacemaker implantation and is associated with high morbidity and mortality. Permanent CIED implantation can have several complications, including lead-related complications; traumatic complications, such as pneumothorax and pericardial effusion; pocket complications; and infection. The incidence of CIED infection in the population greater than one year after implantation is 1.82 per 1000 [20] and is a potential risk for endocarditis. Methicillin-resistant Staphylococcus aureus (MRSA) is the most common cause of CIED infection with other bacterial and fungal agents being less common [21-22]. Treatment is with empiric broad-spectrum antibiotic therapy, which is narrowed following blood and device cultures, with complete device removal in select patients [23].

Dactylitis commonly occurs in patients with a history of psoriatic arthritis, but can also be seen in other conditions, such as reactive arthritis, sarcoidosis, gout, sickle cell disease, and infections [24]. Diagnosis of dactylitis can be made based on clinical assessment, laboratory, radiologic findings, and patient history [25]. In the case of our patient, the diagnosis of dactylitis was made based on the clinical presentation of the patient's fingers as seen in Figure 1, along with right upper extremity CT which showed extensive erosive changes throughout the wrist and hand with associated soft tissue densities and calcifications. 

Risk factors for dactylitis include pre-existing inflammatory conditions such as spondyloarthropathy and also infections that can lead to an inflammatory response, most commonly seen in patients with syphilis and tuberculosis and also seen in patients with Staphylococcal infections, such as in the case of our patient [8, 26].

Imaging modalities used in the evaluation of dactylitis have traditionally included conventional radiographs for bony abnormalities, but more recently ultrasound (US) and MRI have been utilized for better evaluation of soft tissue components and their vasculature [26-27]. Additionally, CT can be used to assess for bone damage, new bone formation, and bony erosions [28]. Digits affected by dactylitis demonstrate greater radiologic progression compared to those without dactylitis [29]. Soft tissue evaluation of dactylitis with US and MRI commonly shows flexor tenosynovitis, and pseudo-tenosynovitis, which is characterized as diffuse inflammation of extra-tendinous soft tissues, enthesitis, and synovitis [30]. Our patient was evaluated with right upper extremity CT which showed extensive erosive changes throughout the wrist and hand with associated soft tissue densities and calcifications. For better characterization of the soft tissues, US or MRI could have been performed to establish baseline imaging to track the progression of the disease.

In patients who develop dactylitis, nonsteroidal anti-inflammatory drugs and local corticosteroid injections are the initial treatment modalities of choice, but their efficacy in reducing the duration of disease has not been supported with controlled trials [25]. Novel biologics (ixekizumab, ustekinumab, and secukinumab), as well as tumor necrosis factor-alpha inhibitors (infliximab, golimumab, adalimumab), have demonstrated a significant reduction in dactylitis when used for 12-14 weeks [26].

Patients with psoriasis are more likely to be colonized by S. aureus on the skin and in the nasal cavity as well as Candida species on the skin and in the oral cavity compared with healthy controls [24, 27]. In our patient, we suspect that the underlying CEID infection, which was secondary to bacterial and fungal colonization, resulted in an acute psoriatic exacerbation that manifested as dactylitis. The patient was treated with an antifungal, antibacterial, and also given non-steroidal anti-inflammatory drugs (NSAIDs) for supportive care and the dactylitis resolved. It is possible that our patient was at increased risk of CIED infection secondary to organism overgrowth associated with his underlying psoriasis.

## Conclusions

Patients with pacemaker placement, total hip/knee arthroplasty, or other invasive procedures are more likely to have a bacterial or fungal infection and possibly dactylitis if they have a history of psoriasis. Physicians should ask if the patient with an infected pacemaker or other invasive procedure has a medical history of psoriasis or other rheumatological diseases, such as systemic lupus erythematosus (SLE) or rheumatoid arthritis.

If the patient has a surgical implantable device in the body, physicians should be aware of colonization of *S. aureus* and *C. lusitaniae* around the device, especially after a revision or an adjustment after the original surgery. The physicians should also look out for skin rash or blistering symptoms.

One needs to be aware of the development of vancomycin infusion reaction if the patient is treated with vancomycin. If the vancomycin infusion reaction is observed, prolong the infusion period to one to two hours, or administer antihistamine concomitantly. Also watch out for psoriatic flare-ups that can occur with a Staphylococcus or Streptococcus infection in patients with rheumatologic conditions, such as psoriasis.
